# Comment on Khanal et al. The Repertoire of Adenovirus in Human Disease: The Innocuous to the Deadly. *Biomedicines* 2018, *6*, 30

**DOI:** 10.3390/biomedicines7010008

**Published:** 2019-01-22

**Authors:** Richard L. Atkinson

**Affiliations:** Virginia Commonwealth University School of Medicine, Richmond, VA 23103, USA; adv36lab@gmail.com; Tel.: +1-804-244-9070

**Keywords:** Adenovirus 36, obesity, etiology

## Abstract

In their comprehensive review on adenoviruses, Khanal et al. omitted obesity as a disease caused by adenovirus 36 (Adv36). Animal studies have shown that experimental infection with Adv36 causes increased adiposity, and human association studies have shown that prior infection with Adv36 is correlated with greater body weight in humans in multiple countries of the world.

## Comment

The paper in *Biomedicines* by Khanal et al. [1] reviewing diseases due to adenoviruses is comprehensive, but the authors left out one disease that is the most prevalent in the US and perhaps the world—obesity. Multiple studies have shown that adenovirus 36 (Adv36) causes obesity when experimentally administered to animals [2,3,4]. Chickens, mice, rats, and monkeys that are infected with Adv36 increase adiposity by 50% to 150%, and 60% to 100% of infected animals become obese [2,3]. Monkeys were shown to have increased body fat by about 60% in 100% of infected animals over a 7-month period [3]. For ethical reasons, humans cannot be experimentally infected, but multiple studies in humans assessing prior infection indicated by the presence of antibodies to Adv36 have concluded that Adv36 is associated with obesity [5,6,7]. The prevalence of Adv36 infection in adults and children with obesity throughout the world averages about 30%, but has a wide range [5,7]. Infection rates in non-obese individuals are lower [5,7]. Since Adv36 was first identified in 1978 and the worldwide epidemic of obesity began about 1980, it may be reasonable to postulate that Adv36 is responsible for about half of the increase in obesity since 1980. Na et al. [4] demonstrated that an anti-Adv36 vaccine prevented obesity in infected mice. Perhaps a large percentage of obesity in the world can be prevented by a vaccine.
